# Consecutive Recovery of Bioactive Substances from *Desmodium canadense* at Different Plant Vegetation Phases by Green Extraction with Supercritical CO_2_ and Increasing Polarity Pressurized Liquids

**DOI:** 10.3390/molecules31030528

**Published:** 2026-02-03

**Authors:** Sana Abbas, Milda Pukalskienė, Laura Jūrienė, Ona Ragažinskienė, Petras Rimantas Venskutonis

**Affiliations:** 1 Department of Food Science and Technology, Kaunas University of Technology, Radvilėnų pl. 19, LT-50254 Kaunas, Lithuania; 2 Kaunas Botanical Garden, Vytautas Magnus University, Ž.E. Žilibero Str. 6, LT-46324 Kaunas, Lithuania

**Keywords:** pressurized liquid extraction, water extract, ethanol extract, acetone extract, antioxidant capacity, UPLC-Q-TOF-MS/MS

## Abstract

This study used high-pressure extraction to obtain antioxidant-rich fractions from *Desmodium canadense* leaves harvested at five vegetation phases (intensive growing to end of blooming) and to evaluate their antioxidant activity and phytochemical profile. Supercritical CO_2_ extraction recovered lipophilic compounds, with the highest yield at massive flowering. The remaining plant material was fractionated by pressurized liquid extraction (PLE) using acetone, ethanol, and water; the highest PLE yield was achieved with water (16.54 g/100 g DW) at the bud formation stage. Antioxidant capacity was measured using total phenolic content (TPC) and ABTS^•+^, CUPRAC, and ORAC assays. Overall, ethanol PLE extracts showed the strongest antioxidant properties: maximum TPC (282.1 mg GAE/gE) and ABTS^•+^ (1010 mg TE/gE) at massive flowering, and highest CUPRAC (853.3 mg TE/gE) and ORAC (1882 mg TE/gE) at bud formation. UPLC-Q-TOF-MS/MS profiling identified 37 compounds, mainly C-glycosyl flavones, flavonol O-glycosides, hydroxycinnamic acid derivatives, and low molecular weight organic acids. Water extracts were rich in low molecular weight organic acids, while acetone and ethanol extracts contained the highest flavonoid levels. Citric acid and vitexin were the most abundant compounds. The findings indicate that *D. canadense* leaves, especially harvested at budding through massive flowering, are a promising source of flavonoid-rich antioxidant extracts for nutraceutical and functional food applications.

## 1. Introduction

Natural antioxidants are considered promising alternatives for the partial replacement of commonly used synthetic antioxidants, such as butylated hydroxytoluene (BHT) and butylated hydroxyanisole (BHA), which have been linked to possible health effects, including liver damage and carcinogenic risks [1]. Consequently, there is growing interest in the finding new, safe, and effective natural antioxidants that can be incorporated into functional foods, nutraceuticals, and cosmeceuticals. In this context, phenolic compounds are among the most significant groups of plant secondary metabolites due to their strong antioxidant properties. Medicinal and edible plants are recognized as major natural sources of these bioactive constituents. However, the efficiency of recovering phenolic compounds is strongly influenced by the extraction strategy employed, including solvent polarity, extraction conditions, and fractionation approaches. *Desmodium* is a genus of flowering plants in the legume family Fabaceae, commonly called tick-trefoil, tick clover, hitchhikers, or beggar. A total of 38,811 occurrences of *Desmodium* Desv. species were recorded worldwide in various published sources [2]. A comprehensive review on the traditional uses in medicine, phytochemistry, and pharmacology of the genus *Desmodium* was published 13 years ago by Ma et al. [3]. About 350 plant species have been used as both feed and herbal medicines, of which about 30 have been phytochemically or pharmacologically investigated. The *Desmodium* plants were known as medicines in China 3000 years ago and, according to records from Chinese and Indian traditional medicine, were used to treat acute diseases such as fever, cold, cough, jaundice, paralysis, edema, asthma, constipation, convulsions, etc. [3].

In total, 212 compounds have been reported in 15 *Desmodium* species, including alkaloids, flavonoids, phenols, phenylpropanoids, terpenoids, steroids, and several volatile constituents. Alkaloids and flavonoids are considered to be the main phytochemical components and may be responsible for most activities, including antioxidant, anti-inflammatory, analgesic, anticancer, anti-depressive, antipyretic, antidepressant, and anti-diabetic exhibited by the plants belonging to this genus [4,5]. Various studies have reported that phenolic and nitrogenous compounds (flavonoids, polyphenols, anthocyanins, carotenoids, and tannins) are mainly responsible for antioxidant activity in *Desmodium* plant extracts [6]. Li et al. [7] reported that 1,3,5,6-tetrahydroxyxanthone is mainly responsible for the antioxidant activity in *D. caudatum*, which is also commonly found in other *Desmodium* plants. Schaftoside, a flavonoid naturally present in *D. styracifolium,* has been reported to have hepatoprotective activity, reducing high-fat diet (HFD)-induced fat accumulation and hepatic ischemia by altering the eicosanoid metabolic pathway in the liver [8].

*Desmodium canadense* (L.) DC., with common names such as showy tick trefoil, or Canada tick trefoil, are primarily found in central Canada and the north-eastern United States. Its leaves are rich in flavonoids; however, their use is limited by the lack of comprehensive studies on plant processing. Soyasaponin Bb’ (soyasaponin III) and the 2,3-dihydro-2,5-dihydroxy-6-methyl-4H-pyran-4-one conjugate of soyasaponin III were identified as major saponin components in the methanolic seed extract of *D. canadense* [9]. Puodziunene et al. [10], isolated and quantified 15 flavonoid glycosides and aglycones (apigenin, luteolin, apigenin-7-O-glycoside, rutin, 2-vicenin, vitexin, isovitexin, vitexin rhamnoside, orientin, homoorientin, quercitrin, quercetin, astragalin, hyperoside, and kaempferol) in reproductive and vegetative parts of *D. canadense* at different phenological stages applying HPLC techniques and reported that the highest content of flavonoids were present in the leaves at ripening phase, and inflorescence from reproductive organs at massive flowering. In another study, they isolated chlorogenic, ferulic, vanillic, caffeic, and 4-hydroxycinnamic acids at various growth phases [11]. To the best of our knowledge, there is no previously published scientific data on the biorefining of *D. canadense* plant material using consecutive high-pressure extraction with increasing-polarity solvents, and on the evaluation of antioxidant activities and the phytochemical composition of the recovered soluble fractions and insoluble residues. Considering health-beneficial properties and previously reported phytochemicals in *D. canadense,* further studies may expand our knowledge and provide valuable information for the broader application of this plant.

The majority of previously published studies employed conventional methods to extract phytochemicals from various botanicals. These extraction methods are often inefficient, require long processing times, and use large amounts of hazardous organic solvents, which must be removed from final extracts to meet safety regulations. They may also lead to the degradation of sensitive compounds and environmental pollution. Therefore, greener approaches such as supercritical CO_2_ extraction (SFE-CO_2_) and pressurized liquid extraction (PLE) have emerged as highly efficient and environmentally friendly alternatives for isolating natural compounds. The main advantages of SFE-CO_2_ extraction are the production of pure extracts (no further solvent removal is required) and environmental friendliness (CO_2_ is non-toxic, non-flammable, and generally recognized as safe (GRAS) solvent). Both SFE-CO_2_ and PLE offer tunable selectivity by adjusting pressure, temperature, and solvent polarity, which allows fractionation of complex plant matrices into phytochemically enriched extracts. Their effectiveness in plant biorefining has been demonstrated in previous studies. For instance, members from our research group have applied high-pressure extraction to *Bergenia crassifolia* roots and leaves, *Solidago virgaurea* leaves, and *Fagopyrum esculentum* flowers, obtaining extracts rich in antioxidants and valuable phytochemicals [12,13,14]. These studies highlighted the versatility and sustainability of green high-pressure extraction methods for the recovery of natural compounds.

This study aimed at the recovery of different polarity soluble fractions of bioactives from *D. canadense* leaves, collected at different plant vegetation phases (intensive growing, bud formation, beginning of flowering, massive flowering, and end of flowering), by using supercritical carbon dioxide (SFE-CO_2_) and pressurized liquid (PLE) extraction techniques, and to evaluate the extracts obtained for their antioxidant properties and phytochemical composition. It is expected that this study will enhance scientific understanding of *D. canadense* by broadening existing phytochemical knowledge and by presenting an effective fractionation method that could facilitate the sustainable utilization of this plant as a source of valuable nutraceutical, functional food, and cosmeceutical ingredients.

## 2. Results and Discussion

### 2.1. Recovery Yield of Non-Polar and Polar Fractions

Botanicals are complex materials that contain substances of varying polarities, a mixture of soluble and insoluble constituents. Therefore, the development of modern fractionation schemes may provide products with unique properties that can be further tailored to meet the requirements of various ingredients for different applications. The primary objective of the first fractionation stage of *D. canadense* was to recover the lipophilic low-polarity components. Such components are of particular interest to the cosmetic industry [15]. The efficiency of SFE-CO_2_ was compared with Soxhlet extraction (hexane) as the standard technique. The SFE-CO_2_ and Soxhlet yields of the *D. canadense* leaves collected at different periods are given in Table 1. SFE-CO_2_ (40 MPa, 50 °C, 360 min) yielded a slightly lower percentage, from 0.96 to 1.4%, compared to Soxhlet extraction, which ranged from 1.18 to 1.54%. However, no statistically significant differences were observed between the two extraction techniques (SFE-CO_2_ and Soxhlet–hexane) at the same growth stage (*p* > 0.05). The highest yields were obtained from the plants harvested at the MF, 1.40 ± 0.05 and 1.54 ± 0.07 g/100 g DW with SFE-CO_2_ and Soxhlet, respectively. The ability to produce solvent-pure extracts using SFE-CO_2_ is advantageous since it eliminates the need for solvent removal after extraction. For instance, the highest permissible solvent residue limits for hexane extracts are strictly regulated [16].

As the results show, there are no significant differences in yields between IG and MF, or between BP, BF, and MF. However, yields at EB differ statistically. This difference may be explained by seasonal changes in plant resource allocation: toward the end of the blooming season (after peak flowering), the plant may begin reallocating resources from leaves to developing organs such as flowers, fruits, and seeds [17]. No published data is available on the extraction of lipophilic compounds from *D. canadense* using SFE-CO_2_ and Soxhlet extraction techniques. Afterward, the SFE-CO2 residues were sequentially fractionated by pressurized liquid extraction (PLE) using increasing-polarity solvents, acetone, ethanol, and water, to isolate higher-polarity phytochemicals with potent antioxidant properties (Table 2).

Acetone, a medium-polarity solvent, is effective at extracting a broad range of semi-polar compounds. The percentage yield of PLE-Ac was in the range of 2.32% (at EB) to 3.48% (at IG) at 70 °C for 45 min (3 cycles 15 min each). The yield of PLE-EtOH ranged between 8.74% and 14.80% (lowest at IG and highest at MF), which was nearly four times higher than the yield obtained with acetone. The yield with water at 140 °C ranged from 12.22% (at EB) to 16.54% (at BP), approximately 5 and 1.44 times that of acetone and ethanol, respectively. Water, as the most polar solvent, is not only capable of dissolving phenolic derivatives but also particularly effective at extracting highly polar primary metabolites, such as sugars, soluble fibers, soluble proteins, and glycosides. These compounds are readily soluble in water and are often co-extracted alongside phenolics, although phenolic recovery is usually enhanced by aqueous alcohol rather than pure water [18].

### 2.2. Proximate Composition

Proximate composition of dried *D. canadense* leaves harvested at different growth periods was determined before and after removing the lipophilic fraction by SFE-CO_2_ (Table 3). Before SFE-CO_2_ the crude protein content was in the range of 18.81–23.73%, minerals 5.08–6.44%, moisture 9.05–8.79%, crude fat 1.17–1.54%, and carbohydrate 59.48–65.68%. After SFE-CO_2_ of lipophilic compounds, the percentage content of other constituents slightly increased; however, in most cases, not significantly. It may be noted that *D. canadense* leaves are exceptionally rich in proteins. The highest protein content was observed in leaves collected during IG; thereafter, it decreased approximately 17–22% and remained stable until EB. Most likely, the proteins after intensive growth undergo biotransformation to produce energy and secondary metabolites. There is no published information on the proximate composition of *D. canadense;* however, the members of its family have been studied for proximate composition analysis. For example, Igboabuchi et al. [19], reported the following proximate values for *D. velutinum* leaves: moisture (10.38 ± 0.01%), ash (21.71 ± 0.02%), crude protein (16.41 ± 0.01%), crude fiber (18.76 ± 0.03%), and carbohydrate (22.10 ± 0.04%). Mohan et al. [4] found 15.12% crude protein, 2.24% fat, 7.38% total ash, 11.85% moisture, and 63.41% carbohydrates in aerial parts of *D. gangeticum.* Baloyi et al. [20], reported that crude protein in *D. uncinatum* leaves decreased from pre-anthesis to post-anthesis, ranging from 150 to 246 g/kg DW, confirming a general trend of protein reduction as *Desmodium* plants transition from vegetative to reproductive stages.

### 2.3. Antioxidant Activity of Solid Residues (QUENCHER Method)

A significant portion of antioxidant compounds may be chemically bound to the solid matrix, potentially leading to an underestimate of the sample’s overall antioxidant capacity. Gökmen et al. [21] developed the QUENCHER (‘QUick, Easy, New, CHEap and Reproducible’) technique to address this problem. It evaluates the antioxidant potential of unextractable solid materials and is compatible with all widely used in vitro antioxidant capacity assessment techniques. The antioxidant activities of the solid samples (powdered leaves before and after SFE-CO_2_) were evaluated using the CUPRAC and ABTS^•+^-scavenging capacity assays, while the total phenolic contents were measured following the Folin–Ciocalteu method (Table 4).

The results in Table 4 showed that TPC values in solid, untreated plant material ranged from 92.12 to 37.06 mg GAE/g DW, while in SFE-CO_2_-defatted material, they ranged from 100.7 to 54.59 mg GAE/g DW. For ABTS^•+^, values varied from 282.9 to 123.9 mg TE/g DW in untreated material, and from 372.9 to 231.7 mg TE/g DW in extracted residue. CUPRAC values ranged from 245.2 to 159.1 mg TE/g DW in untreated samples, and from 264.1 to 180.3 mg TE/g DW in extracted samples. The highest TPC and ABTS^•+^-scavenging values were observed at MF, and the lowest at the EB phases. Although the differences between certain growth phases (IG, BP, and MF) were not substantial, they were statistically significant (*p* < 0.05). Furthermore, the SFE-CO_2_ residues exhibited greater antioxidant activity than the original material. This was probably because the removal of the less-active lipophilic part made the bound antioxidants more readily accessible for reaction in the assays used. Thus, defatted leaves remained 109–147%, 108–131%, and 100–122% of the initial TPC, ABTS^•+^, and CUPRAC values, respectively. As a result, these fractions remain highly valuable for further processing with more polar solvents to obtain antioxidant-rich extracts from *D. canadense* leaves.

### 2.4. Antioxidant Activity of Different Polarity Extracts Obtained by PLE

The in vitro antioxidant capacity of the polar fractions from PLE was evaluated using three single-electron transfer (SET) assays, namely, ABTS^•+^ decolorization, CUPRAC, and TPC, and one hydrogen-atom transfer (HAT) assay, ORAC. These assays were used to assess the antioxidant potential of the dried extracts and their recovery when recalculated on a DW basis, taking into account the extraction yield. For example, a low-yield extract might exhibit more potent antioxidant activity, whereas high yields could result in better recovery of antioxidants from the plant. However, these high-yield extracts may display lower antioxidant capacities because neutral compounds dilute the active constituents. As given in Table 5, the TPC in polar extracts ranged from 90.07 to 282.1 mg GAE/gE, or 11.00 to 38.63 mg GAE/g DW. In general, PLE-EtOH extracts exhibited significantly higher antioxidant capacity values at every growth stage in comparison to acetone and water extracts. The maximum TPC value PLE-EtOH extract was observed at MF, 282.1 mg GAE/gE, and the minimum for EB, 90.07 mg GAE/gE. No studies have reported the TPC of *D. canadense*; however, other *Desmodium* species have been studied for TPC. For example, *D. adscendens* leaves showed approximately 11.15 mg GAE/g DW when extracted with 50% ethanol [22]. While much higher TPC (532.36 mg GAE/gE), levels were obtained by the ethyl acetate fraction of *D. ramosissimum* after solvent fractionation [23]. These differences compared with our results may be attributed to variations in species, plant material, extraction conditions, and solvent polarity, all of which strongly influence phenolic recovery.

**Figure 1:**
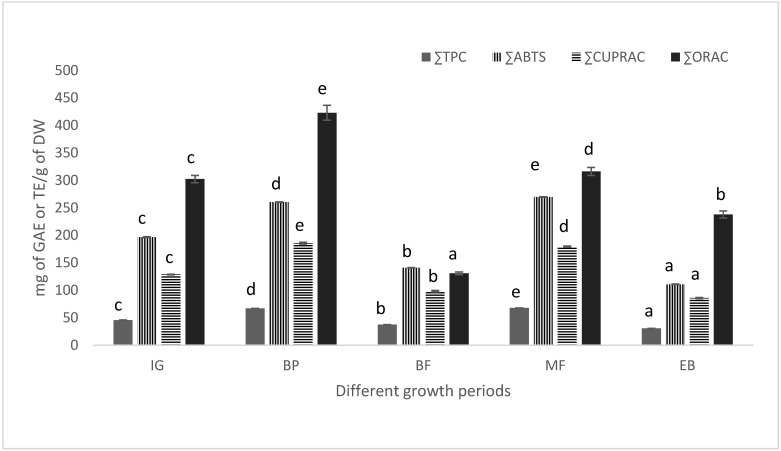
Sum of each antioxidant activity (∑ABTS, ∑CUPRAC, ∑ORAC, expressed as mg TE/g DW) and sum of total phenolic content (∑TPC, expressed as mg GAE/g DW) of PLE extracts extracted with different solvents at different growth periods. Values are mean ± standard deviation (*n* = 3). Different letters above the bars indicate significant differences among growth periods within each assay, as determined by Tukey’s test (95% confidence level).

The ethanol fraction also exhibited the strongest activity for ABTS^•+^ (1010 ± 1 mg TE/gE and 149.5 ± 0.2 mg TE/g DW at MF), CUPRAC (853.3 ± 11.99 mg TE/gE and 108.54 ± 1.53 mg TE/g DW for BP), and ORAC (1882.9 ± 75.50 mg TE/gE and 239.5 ± 9.60 at BP) assays both for extract and DW. In total of 44, 66, 35, 66, and 30 mg of GAE at IG, BP, BF, MF, and MF, respectively, in 1 g of plant material were recovered from three different polarity fractions in PLE. Total of mg TE/g DW 196, 260, 139, 268, 110 (ABTS^•+^), 127, 184, 97, 213, 85 (CUPRAC), and 213, 426, 226, 405, 220 (ORAC) were recovered at IG, BP, BF, MF and EB, respectively, from three different polarity fractions for each growth period in PLE (Figure 1). The highest recovery of TPC and other antioxidants was observed at MF on a per-gram basis. It can be estimated that PLE-EtOH contributed 40–57%, followed by PLE-W (30–44%) and PLE-Ac (11–13%), the major portion of the total phenolic contents.

To the best of our knowledge, there is no published data on the in vitro antioxidant activities of *D. canadense* leaves. This is the first comprehensive report on the systematic evaluation of its total antioxidant capacity. However, studies on other plant species have reported similar observations, in which solvent-derived crude extracts and phenolic-rich fractions showed potent radical-scavenging and reducing capacities that correlate with their phenolic content [23,24,25].

### 2.5. Phytochemical Profile of D. canadense

**Figure 2:**
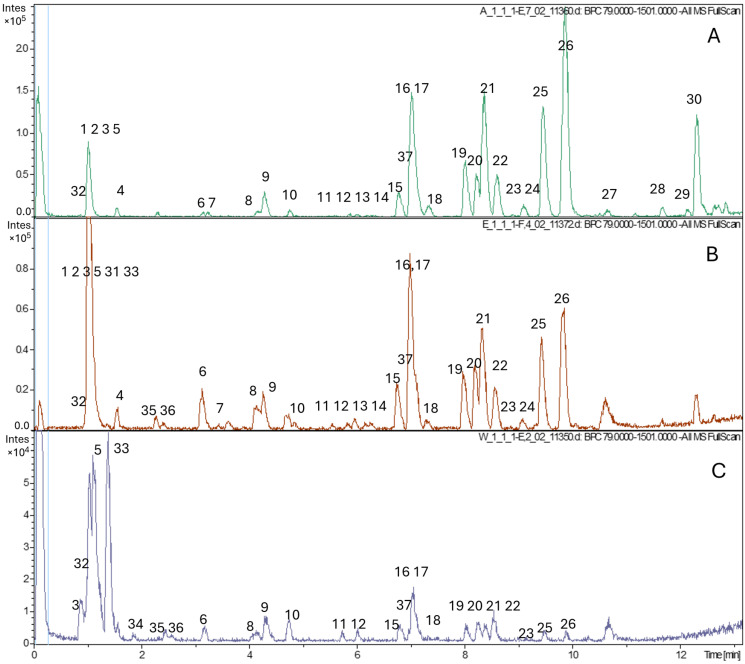
UPLC–QTOF profiles of PLE extracts obtained sequentially with (**A**) acetone, (**B**) ethanol, and (**C**) water.

The comprehensive phytochemical profile of *D. canadense* in PLE extracts of varying polarity was investigated using UPLC-QTOF-MS. A preliminary qualitative phytochemical composition is listed in Table 6, and the chromatograms are shown in Figure 2. The compounds were identified using the following data: chromatographic data, including accurate mass measurements and suggested formulas; previously reported compounds in the *Desmodium* genus; MS/MS fragmentation; mass spectral databases such as Chemspider, Metlin, and Pubchem; and available standards. In total, 37 compounds were identified: 15 by MS/MS fragmentation patterns, 15 by retention time (tR) comparisons with standards, and the remaining by interpreting mass spectra using previously reported data. Compound **1** found in acetone and ethanol extracts was tentatively identified as a monosaccharide giving molecular ion [M – H]^−^ with molecular formula *m*/*z* 179.0563. Compounds **2** ([M – H], *m*/*z* 341.1093) and 31 ([M – H]^−^, *m*/*z* 683.2254) were tentatively identified as structures exhibiting MS features that are characteristic of deprotonated oligosaccharide ion dimers. However, no matching compounds were found in the literature, so no specific name could be assigned. Compound **3** ([M – H]^−^, *m*/*z* 387.1146) was identified in all analyzed extracts as an unknown disaccharide with different hexose units. This is confirmed by Wan et al. [26], who investigated the fragmentation pathways of glucose-containing disaccharides using negative-mode ESI-MS/MS and reported spectra of disaccharides observed in our extracts. Compound **4** gave [M – H]^−^, *m*/*z* 117.0191, the molecular formula C_4_H_5_O_4_ assigned the name succinic acid, and was present in all three extracts. This compound was confirmed using an authentic standard.

Compound **9**, with the molecular formula C_15_H_13_O_6_ and an ion peak at *m*/*z* 289.0718, was identified as catechin by comparison with the standard. Additionally, the characteristic fragment ion given by MS/MS at *m*/*z*, 245.0809 corresponding to molecular formula C_14_H_13_O_4_, could be due to the loss of a -CH_2_-CHOH group. According to some studies, compound **10** was identified as chlorogenic acid [25,27]. This compound showed [M – H]^−^ at *m*/*z* 353.0875 with the molecular formula C_16_H_17_O_9_ MS/MS fragmentation pattern gave three fragments, *m*/*z* 135.0450, *m*/*z* 179.0350, and 191.0560. The *m*/*z* 135.0450 with formula C_8_H_7_O_2_ corresponds to the breakdown of the caffeic acid fragment with the loss of CO_2_, *m*/*z* 179.0350 corresponds to the deprotonated caffeic acid (C_9_H_7_O_4_) portion, and 191.0560 indicates the deprotonated quinic acid (C_7_H_11_O_6_). The authenticity of this compound was also confirmed by using a reference standard.

Compounds **16**, **18**, **23**, **26**, **27**, **21**, **22**, and **20** were identified based on their molecular ions and comparison with reference standards. Compounds **16**, **18**, **23**, **26**, and **27**, all sharing the molecular formula [C_21_H_19_O_11_]^−^ and the same mass ([M – H]^−^, *m*/*z* 447.093), as well as compounds **21** [M – H]^−^, *m*/*z* 431.0991), **22** [M – H]^−^, *m*/*z* 463.0877), and **20** ([M – H]^−^, *m*/*z* 609.1461) have previously been reported in *D. canadense* [28]. Based on comparison with reference standards, these compounds were assigned as homoorientin (**16**), orientin (**18**), luteolin-7-O-glucoside (**23**), quercitrin (**26**), astragalin (**27**), vitexin (**21**), quercetin 3-O-glucoside (**22**), and rutin (**20**). Homoorientin, astragalin, vitexin, hyperoside, and rutin were further confirmed by their characteristic MS/MS fragmentation patterns. Homoorientin produced the ion [M – H]^−^ at *m*/*z* 447.0933, while the fragment at *m*/*z* 429.0824 [M – H – 18]^−^ resulted from the loss of a water molecule. The aglycone fragment at *m*/*z* 285.0406 [M – H – 162]^−^ indicated the loss of a hexose unit, and additional ions at *m*/*z* 357.0620 [M –H – 90]^−^ and 327.0513 [M – H–120]^−^ corresponded to the loss of C_3_H_6_O_3_ and C_4_H_8_O_4_ moieties, respectively, through collision-induced dissociation of the glycone portions [29]. Vitexin (**21**) exhibited a parent ion at *m*/*z* 431.0983 and two MS/MS fragmentation ions at *m*/*z* 341.0664 and 311.0559, corresponding to the loss of [M – H – 90]^−^ and [M – H – 120]^−^ moieties, respectively, which are consistent with the molecular formulas C_18_H_13_O_7_ and C_17_H_11_O_6_ [30]. Rutin (**20**) showed a parent ion [M – H]^−^ at *m*/*z* 609.1461 and produced a fragment ion at *m*/*z* 300.0273 in the MS/MS spectra, corresponding to the deprotonated quercetin aglycone. According to Ndou et al. [31], this fragmentation resulted from cleavage of the glycosidic bond and loss of the sugar units—rhamnose and glucose. Astragalin (**27**) exhibited a parent ion [M – H]^−^ at *m*/*z* 447.0933 and two MS/MS fragment ions at *m*/*z* 285.0389 and 151.0033 (C_7_H_3_O_4_), respectively. The fragment at *m*/*z* 285.0389 corresponds to the deprotonated kaempferol aglycone (C_15_H_9_O_6_), formed by the loss of a glucose unit (162 Da); the fragment at *m*/*z* 151.0033 is a characteristic ion of the kaempferol backbone resulting from further aglycone fragmentation. The results obtained in this study are in agreement with those reported in previous studies [32,33].

The parent ion peak [M – H]^−^ for compound **17** was observed at *m*/*z* 563.141 with molecular formula C_26_H_27_O_14_ and fragmented into several characteristic product ions. The fragment at *m*/*z* 545.1294 [M – H – 18]^−^ corresponded to the loss of a water molecule, while *m*/*z* 473.1086 [M – H–90]^−^ suggested the removal of a sugar moiety, typically associated with glycosylated flavones. Further fragmentation of the sugar unit generated ions at *m*/*z* 443.0985 [M – H – 120]^−^, *m*/*z* 383.0771 [(M – H) – 120 – 60]^−^, and *m*/*z* 353.0664 [(M – H) – 120 – 90]^−^. Although the fragmentation pattern is consistent with C-glycosyl flavones reported in the literature [33,34], the available data were insufficient for unambiguous structural elucidation; therefore, this peak was classified as an unidentified compound.

Compound **24** was identified as kaempferol-3-O-rutinoside. Its structure was confirmed using the ChemSpider database based on its exact mass and formula [M – H], *m*/*z* 593.1511; C_27_H_29_O_15_. MS/MS fragmentation data yielded the fragment ion at *m*/*z* 285.0405, indicating the deprotonated kaempferol moiety; 593–285 leaves a remainder of 308, which suggests a rutinose residue, further supporting this annotation. These results are also supported by the identification of kaempferol-3-O-rutinoside reported by other authors [35]. Compound **19** showed a parent ion [M – H]^−^ at *m*/*z* 563.1415 with molecular formula C_26_H_27_O_14_, generating two MS/MS fragments at 413.0879 (C_21_H_17_O_9_) and 293.0455 (C_17_H_9_O_5_). According to Zhou et al. [34], an ion with the same [M – H]^−^ at *m*/*z* 563 and a similar MS/MS pattern was tentatively assigned as an apigenin xyloside, with two possible isomeric structures proposed: apigenin-6-C-glucosyl-2″-O-xyloside and apigenin-8-C-glucosyl-2″-O-xyloside. On this basis, compound 19 in the present study can be tentatively annotated as an apigenin xyloside (apigenin C-glucosyl-O-xyloside). However, due to the possibility of positional isomers and the absence of an authentic standard, this identification should still be regarded as tentative.

Compound **25** with a deprotonated ion at *m*/*z* 593.1513 [M – H]^−^ and molecular formula C_27_H_29_O_15_ was identified as a C-glycosyl type of flavone, based on literature data on *Desmodium* species [36]. Compound **15** showed a deprotonated molecular ion at *m*/*z* 579.1359 [M – H]^−^, consistent with the elemental composition C_26_H_27_O_15_. In the MS/MS spectrum, the most intense product ion was observed at *m*/*z* 459.0931 [M – H – 120]^−^, which can be attributed to a neutral loss of 120 Da from the precursor ion, while a further fragment at *m*/*z* 429.0823 [M – H –150]^−^ likely reflects an additional sugar-related cleavage. These neutral losses suggest the presence of a di-glycosylated flavonoid structure. For this reason, compound 15 could be reported as an unidentified flavonoid diglycoside.

Compounds **8**, **12**, **13**, and **14** showed the same deprotonated molecular ion at *m*/*z* 337.093 [M – H]^−^ and elemental composition C_16_H_17_O_8_. According to Barros et al. [37], this ion can be tentatively assigned to coumaroylquinic acid isomers, most likely corresponding to 3-O-, 4-O-, and 5-O-coumaroylquinic acids. Compound **6** exhibited the same molecular formula (C_16_H_17_O_9_) and mass (*m*/*z* 353.1130) as chlorogenic acid and was also confirmed by comparison with the standard. Compounds **7** and **11**, with deprotonated ions at *m*/*z* 371.1056 and 431.1915 [M H]^−^ and molecular formulas C_16_H_19_O_10_ and C_20_H_31_O_10_ were not identified due to insufficient information for precise identification.

Compounds **29** and **30** were tentatively identified as the flavonol quercetin and the flavone luteolin, respectively. In negative ionization mode, these late-eluting features at R_t_ 11.7 and 12.3 min showed deprotonated molecular ions at *m*/*z* 301.0353 [M – H]^−^ and 285.0401 [M – H]^−^, corresponding to molecular formula C_15_H_9_O_7_ and C_15_H_9_O_6_ in agreement with ChemSpider records for quercetin and luteolin, respectively. Mono and dihydroxybenzoic acids are commonly reported bioactive substances of *Desmodium* species; therefore, based on the results of Li et al. [38,39], compounds **34**–**36** were identified as gentisic acid (2,5-dihydroxybenzoic acid; [M – H]^−^ *m*/*z* 153.0190, C_7_H_5_O_4_); gentisoyl O-hexoside (gentisic-acid glucoside; [M – H]^−^ *m*/*z* 315.0794, C_13_H_15_O_9_), and gentiopicroside (gentiopicrin) ([M – H]^−^ *m*/*z* 355.1030, C_16_H_19_O_9_), respectively. Fernández et al. [40], described the impact of dihydroxybenzoic acids, for instance, gentisic acid, a naturally occurring phenolic acid with reported antioxidant and anti-inflammatory activities, while gentisoyl glucoside represents its glycosylated derivative, functioning as a defensive phytochemical metabolite and also known as a product of aspirin metabolism. In addition, the gentisoyl moiety can be incorporated into larger molecular structures, thereby contributing to diverse biological roles.

Compounds (**5**) [M – H]^−^, *m*/*z* 133.0141, C_4_H_5_O_5_; (**32**) [M – H]^−^, *m*/*z* 191.0194, C_6_H_7_O_7_; (**33**) [M – H]^−^, *m*/*z* 191.0567, C_7_H_11_O_6_; and (**37**) [M – H]^−^, *m*/*z* 163.0473, C_9_H_7_O_3_ were identified as malic acid, citric acid, quinic acid, and *p*-coumaric acid, respectively, based on a comparison with the authentic standards. Compound **36** ([M – H]^−^, *m*/*z* 355.1030, suggested formula C_16_H_19_O_9_) was identified as gentiopicrin (belongs to iridoids) based on the formula given by ChemSpider.

### 2.6. Quantitative Composition of D. canadense Leaves PLE Extracts

The identified compounds were quantified, and their contents are reported as mg per 100 g of dry matter (mg/100 g DW; Table 7). The concentrations of citric, quinic, malic, *p*-coumaric, and chlorogenic acids were consistently higher in water extracts in all growth stages compared to ethanolic and acetone extracts. Citric acid was found to be most abundant (627.6–152.1 mg/100 g DW), followed by malic acid (352.3–142.5 mg/100 g DW), quinic acid (78.09–41.60 mg/100 g DW), chrologenic acid (11.35–4.12mg/100 g DW), and *p*-coumaric acid (10.35–5.42 mg/100 g DW) in water extracts. The maximum levels of these compounds were observed at different growth stages: citric acid during massive flowering, quinic acid during budding, malic acid at the beginning of flowering, and *p*-coumaric acid again during massive flowering. An exception was chlorogenic acid, which reached its highest concentration in the ethanolic extract at the intensive growth stage (16.56 mg/100 g DW), while at the other stages it was more abundant in the water extracts. The general extraction trend followed the solvent polarity order of PLE (acetone < ethanol < water). Since organic acids are highly polar and soluble in water, they can be recovered more efficiently than moderately polar ethanol or less polar acetone.

To our knowledge, the organic acids (citric, malic, and quinic) in *D. canadense* have been identified for the first time. However, Puodzhyunene et al. [11] quantified phenolic acids in *D. canadense* herbs and reported chlorogenic acid at 0.120 mg/g; moreover, they noted that the highest levels of acids were found during the budding and flowering stages using 50% methanol. Comparable organic-acid and phenolic-acid profiles have also been described in related *Desmodium* species. The content of *p*-coumaric acid has been quantified in *D. velutinum* in both the aqueous–methanol stem fraction and the ethyl acetate fraction of methanolic root extracts, with reported concentrations of 9.61 µg/100 g and 1.74 µg/100 g, respectively [41,42]. These studies show that *D. canadense* leaves have a higher concentration of organic acids than other materials in the Fabaceae family, but hydroxycinnamoyl-quinic derivatives, like chlorogenic acid, are found at lower to moderate concentrations than in other *Desmodium* species.

Regarding flavonols (rutin, quercitrin, and quercetin 3-glucoside), rutin and quercetin 3-glucoside showed the highest concentrations in ethanolic extracts at all growth stages, whereas quercitrin was most abundant in acetone extracts. Water extracts contained the lowest levels of all flavonols. In general, BF showed the highest flavonol concentrations across all extracts (quercitrin, 131.8 mg/100 g DW; rutin, 108.4 mg/100 g DW; and quercetin-3-glucoside, 36.41 mg/100 g DW), which then dropped at EB. Among the quantified flavones, vitexin was consistently the most abundant compared with orientin and luteolin-7-O-glucoside in all extracts, and the highest levels were detected in acetone extracts, ranging from 120.4 to 448.2 mg/100 g DW (lowest at EB; highest during the IG period). Orientin and luteolin-7-O-glucoside concentrations were comparable in both acetone and ethanol extracts and were highest at BP. Catechin (flavanol) was most abundant in ethanolic extracts (32.64–56.01 mg/100 g DW; highest at BP) and lowest in acetone (10.13–23.75 mg/100 g DW; highest at MF). Puodziunene et al. [43] quantified 15 flavonoids in *D. canadense* leaves and found that their total content was highest at BP. At this stage, the profile was dominated by C-glycosyl flavones, with orientin and homoorientin as the major constituents (6236 µg/g of sample), followed by, vitexin (1717 µg/g of sample), isovitexin (1767 µg/g of sample), and rutin (925.7 µg/g of sample), while quercitrin was present only in minor amounts. Nkwocha et al. [42] quantified individual phenolics in the ethyl acetate fraction of a methanolic root extract of *D. velutinum*, with chlorogenic acid 0.6052 µg/g, catechin 1.1424 µg/g, and rutin hydrate 0.1253 µg/g. In contrast, the corresponding compounds in *D. canadense* leaf extracts exhibit roughly 100–1000-fold higher concentrations of these phenolics than those in *D. velutinum* roots under the reported conditions, likely reflecting differences in species, plant organ, and extraction procedures. Many factors can explain the observed changes in concentrations of different compounds: solvent/solute compatibility, structural differences between C-glycosides (vitexin, orientin) and the more hydrophilic O-glycoside luteolin-7-O-glucoside, and phenology-driven changes in tissue composition [44,45].

**Figure 3:**
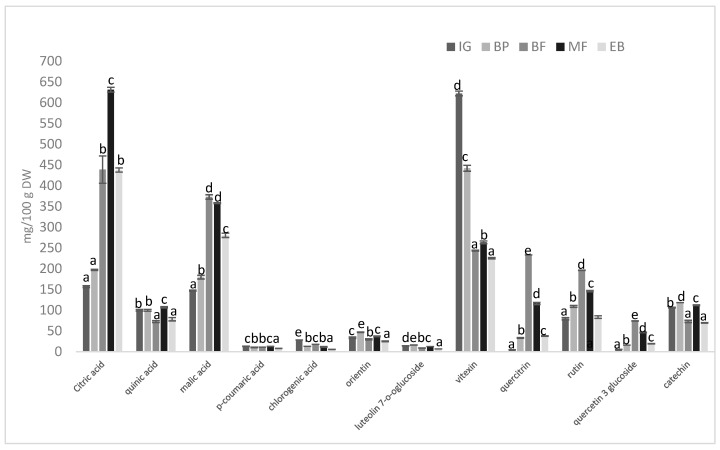
Sum of each compound extracted with acetone, ethanol, and water (expressed as mg TE/100 g DW) at each growth stage. Analyses were performed with PLE extracts at five growth periods, including IG: intensive growth, BP: budding phase, BF: beginning of flowering, MF: massive flowering, and EB: end of blooming. Different letters above the bars indicate significant differences among growth periods within each compound, as determined by Tukey’s test at the 95% confidence interval.

The total content of each compound, calculated as the sum of acetone, ethanol, and water extracts at a given vegetation phase, is presented in Figure 3 and in a separate table in the Supplementary Material (Table S1), which lists the summed concentrations. The data show how individual compounds in *D. canadense* leaves vary across the different growth stages. Overall, citric acid and vitexin are the most abundant constituents, reaching about 600–650 mg/100 g DW at MF and IG, respectively. Malic acid, quercitrin, rutin, and catechin form a second group with intermediate levels, whereas quinic acid, orientin, luteolin-7-O-glucoside, and quercetin-3-glucoside occur in lower amounts. For most compounds, the lowest contents were observed at IG and at EB, while BF and especially MF exhibited significant quantities, indicating that many organic acids and flavonoids accumulate around the flowering period. Phenolic acids and flavonoids are synthesized via the phenylpropanoid pathway, whose activity is regulated by plant development and environmental stresses; therefore, their concentrations change throughout the developmental phases [46,47]. Because these compounds protect young leaves, buds, and flowers against herbivores, pathogens, and UV radiation, and also contribute to floral color, their biosynthesis naturally increases during reproductive development and then declines as resources are redirected to seed formation and tissue aging [48,49]. Therefore, high concentrations at the budding, early flowering, and full-flowering stages are expected, and *D. canadense* should be collected during these periods when flavonoid accumulation is at the highest level.

## 3. Materials and Methods

### 3.1. Plant Material

*D. canadense* (L.) DC. plants were grown in the Botanical Garden of Vytautas Magnus University (54°52′14″ N/23°54′40″ E, Kaunas, Lithuania) and collected at five different growing stages in May–August 2022: intensive growing (IG), bud formation (BF), beginning of flowering (BF), massive flowering (MF) and end of blooming (EB). The plants were sorted into different anatomical parts (leaves, stems, and flowers), and the leaves were used in experimental studies. Plant samples were air-dried in a dark, well-ventilated space at room temperature (20–25 °C) and ground to a 0.2 mm particle size using a high-speed centrifugal mill (Retsch ZM200, Retsch, Haan, Germany). The ground material was kept in tightly sealed dark glass jars in a well-ventilated storage area to prevent microbial and moisture contamination until further extraction and fractionation.

### 3.2. Chemicals and Solvents

CO_2_ and Nitrogen gas (99.9%) were purchased from Gaschema (Jonava, Lithuania), ethanol (96.6%) of agricultural origin from MV Group Production (Kaunas, Lithuania), Folin–Ciocalteu’s phenolic reagent (Bornem, Belgium), Sodium carbonate (98%, anhydrous) from RPL (Grauwmeen, Belgium), gallic acid (99%) from Sigma-Aldrich (Steinhein, Germany), 6-hydroxy, 2,5,7,8-tetramethylchroman-2-carboxylic acid (Trolox, ≥97%), 2,2-azino-bis (3-ethylbenzothiazoline-6-sulphonic acid), diammonium salt (ABTS), sodium chloride (NaCl), potassium chloride (KCl), potassium persulfate (K_2_S_2_O_8_), sodium hydrogen phosphate (Na_2_HPO_4_), potassium dihydrogen phosphate (KH_2_PO_4_), and Neocuproine were obtained from Sigma-Aldrich (St. Luois, MO, USA). Cellulose (microcrystalline powder 20um), 2,2-azobis (2-amidino-propane) dihydrochloride (AAPH), fluorescein sodium salt, copper(II) chloride dihydrate (CuCl_2_ · H_2_O), ammonium acetate (CH_3_COONH_4_), (UAB “Eurochemicals”, Vilnius, Lithuania), diatomaceous earth 100% SiO_2_ (Dionex Corporation, Sunnyvale, CA, USA), orientin and luteolin-7-O-glucoside were obtained from Toronto Research Chemicals (TRC, Vaughan, ON, Canada), and quercetin 3-glucoside and catechin from PhytoLab, (Dutendorfer, Germany); *p*-coumaric acid, quercitrin and vitexin were obtained from ChemCruz, (Finell St., Dallas, TX, USA). All reagents and solvents were of analytical grade.

### 3.3. Determination of Proximate Composition

The composition of *D. canadense* was determined by the following methods of Association of Official Analytical Chemists (AOAC, 2019): protein content by the Kjeldahl method applying a conversion factor of 6.25 (AOAC 960.52); moisture by drying 3 g of ground leaves at 105 °C to constant weight (AOAC 925.10); and ash by mineralizing in a muffle furnace F-A1730 Thermolyne Corp. (Dubuque, IA, USA) at 500 °C (AOAC 900.02); crude fat by Soxhlet extraction with hexane for 3 h (AOAC 920.39) and carbohydrate content was calculated by deducting the proportion of the identified components from 100%. All analyses were conducted in triplicate.

### 3.4. Recovery of Non-Polar Fraction via SFE-CO_2_

Supercritical fluid extraction with CO_2_ (SFE–CO_2_) was applied to extract lipophilic fraction from *D. canadense* leaves in a Helix extractor (Applied Separation, Allentown, PA, USA) equipped with 50 mL stainless steel extraction cell (320 mm length and 14 mm diameter), which was filled with 20 g of powdered plant material and closed with the cotton plugs on both ends. The extraction vessel was placed inside the heating jacket to maintain its temperature. The process was performed at the following parameters: 40 MPa pressure, 50 °C temperature, 2 L/min flow rate (regulated manually using the micrometer valve knob in the system), and 120 min total time, including 10 min of static extraction. These parameters were chosen because they yielded high yields in earlier reports on a variety of botanicals [50,51]. The extracts were collected in dark glass vials, which were kept open until constant weight to remove CO_2_ residues. The extracts were stored at −18 °C until further analysis. The extraction yield of the lipophilic fraction was determined gravimetrically (±0.001 g) and expressed in g/100 g DW.

### 3.5. Valorization of Residual Biomass by PLE

PLE of SFE–CO_2_ residues were performed in an ASE 350 apparatus (Thermo Scientific Dionex, Sunnyvale, CA USA) following the method of Syrpas et al. [51] with slight modifications. The samples (5 g) were mixed with 5 g of diatomaceous earth and placed in a 34 mL stainless-steel extraction cell fitted with filter papers, Glass Fiber-(X)-Cellulose (Dionex Corporation, Sunnyvale, CA, USA) at both ends. The extraction was carried out at 10.3 MPa pressure, sequentially using increasing-polarity solvents: acetone (70 °C), ethanol (70 °C), and water (140 °C). Nitrogen purge time was 120 s, and the cell flush volume was 100%. The pre-heat period was 5 min, and extraction was performed in 3 cycles of 15 min each. Acetone and ethanol were evaporated in a Büchi rotary evaporator, while water was removed by freeze-drying. The yield was expressed as g/100 g DW, and the extracts were stored in dark glass vials at −18 °C until further analysis.

### 3.6. In Vitro Antioxidant Capacity of Extracts and Solid Residue

The ORAC assay evaluated antioxidant activity [52], measuring the inhibition of peroxyl radical-induced fluorescein decay by antioxidants and reporting results as Trolox equivalents. The ABTS^•+^-decolorization assay [53] assessed the ability of samples to quench the ABTS^•+^, with results expressed as milligrams Trolox equivalents per gram of extract (mg TE/gE) and plant dry weight (mg TE/g DW). The CUPRAC assay [54] determined the reducing capacity of antioxidants toward Cu(II)-neocuproine complexes, expressed as mg TE/gE and mg TE/gDW. Both solution-based and QUENCHER procedures were applied; full details are provided in the Supplementary Materials.

### 3.7. Total Phenolic Content (TPC)

TPC was determined using the Folin–Ciocalteu (FC) method [55], based on the reduction in the (FC) reagent by phenolic compounds under alkaline conditions, and the results were expressed as milligrams gallic acid equivalents per gram of extract (mg GAE/gE) and plant dry weight (mg GAE/g DW). Both solution-based and QUENCHER approaches were used; full procedure descriptions are provided in the Supplementary Materials.

### 3.8. Qualitative and Quantitative Characterization of Extracts by Ultra-Performance Liquid Chromatography–Electrospray Ionization–Quadrupole–Time-of-Flight–Mass Spectrometry (UPLC/ESI–QTOF–MS)

Chromatographic analysis of the extracts was performed using a Waters Acquity system (Waters, Milford, CT, USA) coupled to a hybrid quadrupole time-of-flight mass spectrometer (Q-TOF) (Bruker Daltonics, Bremen, Germany). Separation of analytes was carried out on a RP analytical column Acquity BEH C18, 2.1 × 100 mm, with a particle size of 1.7 μm (Waters, Milford, CT, USA), maintained at 40 °C. The mobile phase consisted of eluents: A 0.1% formic acid (*v*/*v*), and B 100% acetonitrile. The total elution time was 17 min. The following gradient program was applied: 0 min—5% B; 0–9 min—5–20% B; 9–12 min—20–50% B; 12–15 min—50–100% B; and 16–17 min 5%. The mobile-phase flow rate was 0.4 mL/min, and the injection volume was 1 µL. Compounds eluting from the column were detected using a diode array detector (DAD; Waters, Milford, CT, USA) in the wavelength range of 100–500 nm. The Q-TOF mass detector was operated in negative electrospray ionization mode, with parameters controlled by HyStar 3.2 software. Ionization was performed at +4000 V, the fragmentation cell voltage was set to 8 eV, and nitrogen was used as both the nebulizing gas (2 bar) and the drying gas, with a flow rate of 10 L/min. By combining full-scan and MS/MS modes, accurate molecular formulas of the compounds were calculated in the m/z range of 100–1500 at a scan rate of 2.5 Hz. The MS/MS mode was used to confirm compounds that could not be unambiguously identified. Identification of chromatographic peaks was based on analyte and reference standard mass spectra, retention time, MS/MS fragmentation patterns, and comparison with literature data. For the identification of unknown compounds, the MS/MS mode was applied with a fragmentation cell voltage of 30 eV. Quantitative analysis was performed using external standards. Calibration curves were constructed using standard solutions at different concentrations (1–50 µg/mL): vitexin (y = 32,012.02x + 18,350.82; R^2^ = 0.999), quinic acid (y = 22,017.10x + 33,336.61; R^2^ = 0.995), quercetin 3-glucoside (y = 26,255.87x + 57,214.53; R = 0.992), luteolin-7-O-glucoside (y = 19,619.70x + 72,026.08; R^2^ = 0.986), malic acid (y = 15,434.33x + 7056.96; R^2^ = 0.997), and rutin (y = 17,344.11x + 12,131.32; R^2^ = 0.999), chlorogenic acid (y = 20,363.88x + 67,957.23; R^2^ = 0.998 ), quercitrin (y = 34,370.55x + 41,027.90; R^2^ = 0.999), catechin (y = 27,420.86x + 37,833.19; R^2^ = 0.999), citric acid (y = 13,768.01x − 7725.03; R^2^ = 0.999), orientin (y = 26,251.36x + 10,761.23; R^2^ = 0.998), and *p*-coumaric acid (y = 46,054.81x + 10,444.22; R^2^ = 0.999). The obtained calibration curves demonstrated the relationship between peak areas and the amount of standards. Compound concentrations were calculated from linear regressions of peak areas generated with QuantAnalysis 2.0 software and expressed as mg/100 g DW extract.

### 3.9. Statistical Analysis

All analyses were performed in triplicate and represented as means ± standard deviations (SD). Mean values and standard deviations were calculated using MS Excel 2016. Significant differences between means were determined using a one-way ANOVA in Statgraphics 19-X64. Tukey’s HSD (honestly significant difference) was applied to determine significant differences among the treatments at the 95% confidence interval.

## 4. Conclusions

In this study, an innovative green biorefinery approach was developed to valorize *D. canadense* leaves collected at various growth stages. Supercritical CO_2_ extraction removed a substantial portion of non-polar components, thereby improving the subsequent recovery of polar compounds. Pressurized liquid extraction with solvents of increasing polarity—particularly ethanol—yielded phenolic-rich fractions with the highest total phenolic content and the highest antioxidant potential in the ABTS^•+^ at massive flowering, whereas in the CUPRAC and ORAC assays it was at the budding phase.

UPLC analysis revealed a total of 37 phenolic and related compounds, with the majority of flavonoids (mainly C-glycosyl flavones and flavonol O-glycosides, such as quercetin, kaempferol, orientin, and vitexin derivatives) and hydroxycinnamic acid derivatives (including chlorogenic, caffeoylquinic, and coumaroyl-quinic acids), along with a low-molecular-weight organic acids (e.g., quinic, citric, malic, and succinic acids) and a smaller number of simple sugars. Water extracts contained the highest amounts of organic acids (citric, malic, and quinic). In contrast, acetone and ethanol extracts were particularly rich in flavonoids, especially vitexin and rutin, confirming that *D. canadense* is a valuable source of phenolics with high antioxidant potential. Overall, the results demonstrate that *D. canadense* leaves, particularly those harvested at the budding, beginning, and massive flowering stages, can be efficiently upgraded into phenolic rich antioxidant ingredients using integrated supercritical CO_2_ and pressurized liquid extraction. This sustainable biorefinery approach provides a promising basis for the development of natural antioxidant formulations for food, cosmetic, or nutraceutical applications and can be adapted to other underutilized plant materials. Despite encouraging results this work lacks practical application of antioxidant rich extracts and future investigations should include extensive profiling of polar and semi-polar substances, precise measurement of critical indicators, and validation of biological effects through cell-based or in vivo models to enhance practical applicability.

## Figures and Tables

**Table 1:** The yield (g/100 g DW) of lipophilic fraction in *D. canadense* leaves at different growth stages was obtained by Soxhlet extraction with hexane (Sox-Hex) and supercritical fluid extraction with carbon dioxide (SFE-CO_2_).

Sample	Intensive Growth	Budding Phase	Beginning of Flowering	Massive Flowering	End of Blooming
SFE-CO_2_	_A_ 1.34 ± 0.05 ^c^	_A_ 1.17 ± 0.09 ^b^	_A_ 1.16 ± 0.01 ^b^	_A_ 1.40 ± 0.04 ^c^	_A_ 0.96 ± 0.01 ^a^
Sox-Hex	_A_ 1.41 ± 0.14 ^bc^	_A_ 1.17 ± 0.06 ^a^	_A_ 1.25 ± 0.02 ^ab^	_A_ 1.54 ± 0.07 ^c^	_A_ 1.41 ± 0.01 ^bc^

Values with different lowercase letters in the same row differ significantly between growth stages, and values with different uppercase letters in the same column differ significantly between extraction techniques (Tukey’s test, mean ± SD, significance level 95%).

**Table 2:** The yield (g/100 g DW) of *D. canadense* leaf fractions at different growth stages obtained by PLE using different polarity solvents, acetone (Ac) ethanol (EtOH), and water (W).

Sample	Intensive Growth	Budding Phase	Beginning of Flowering	Massive Flowering	End of Blooming
PLE-Ac	_A_ 3.48 ± 0.09 ^c^	_A_ 3.13 ± 0.31 ^c^	_A_ 2.92 ± 0.10 ^b^	_A_ 3.43 ± 0.08 ^c^	_A_ 2.32 ± 0.09 ^a^
PLE-EtOH	_B_ 8.74 ± 2.05 ^a^	_B_ 12.72 ± 0.22 ^c^	_B_ 9.12 ± 0.22 ^b^	_B_ 14.80 ± 0.67 ^d^	_B_ 9.44 ± 0.39 ^b^
PLE-W	_C_ 15.94 ± 1.32 ^b^	_C_ 16.54 ± 0.11 ^b^	_C_ 13.89 ± 0.47 ^a^	_C_ 16.17 ± 0.41 ^b^	_C_ 12.22 ± 2.92 ^a^

The results are expressed as mean ± standard deviation of three replicates ^a,b,c,d^ Different lower-case letters in the same row indicate statistical differences between growth stages. _A,B,C_ Different uppercase letters within the same column indicate statistical differences between different polarity fractions (Tukey’s test at a significant level 95%).

**Table 3:** Proximate composition analysis of *D. canadense* leaves at different growth stages before and after removal of lipophilic fraction.

Botanical Period	Protein, %	Mineral, %	Moisture, %	Crude Fat, %	Carbohydrates, %
Before CO_2_ extraction					
Intensive growth	23.73 ± 0.89 ^c^	6.44 ± 0.06 ^c^	9.05 ± 0.05 ^a^	1.41 ± 0.14 ^bc^	59.48 ± 0.77 ^a^
Budding phase	19.03 ± 0.18 ^ab^	5.08 ± 0.07 ^a^	9.02 ± 0.12 ^a^	1.17 ± 0.06 ^a^	65.68 ± 0.06 ^c^
Beginning of flowering	18.72 ± 0.24 ^ab^	5.45 ± 0.06 ^a^	8.95 ± 0.09 ^a^	1.25 ± 0.02 ^ab^	65.57 ± 0.17 ^bc^
Massive flowering	18.57 ± 0.03 ^a^	5.54 ± 0.06 ^b^	8.80 ± 0.25 ^a^	1.54 ± 0.07 ^c^	65.53 ± 0.25 ^bc^
End of blooming	19.81 ± 0.18 ^b^	5.09 ± 0.22 ^b^	8.79 ± 0.11 ^a^	1.41 ± 0.01 ^bc^	64.97 ± 0.05 ^b^
After CO_2_ extraction					
Intensive growth	24.05 ± 0.90 ^c^*	6.53 ± 0.06 ^c^*	9.17 ± 0.06 ^a^*	0.11 ± 0.14 ^b^*	59.02 ± 0.50 ^a^*
Budding phase	19.25 ± 0.18 ^ab^*	5.14 ± 0.07 ^b^*	9.13 ± 0.11 ^a^*	0.04 ± 0.13 ^a^*	65.89 ± 0.06 ^c^*
Beginning of flowering	18.94 ± 0.25 ^ab^*	5.52 ± 0.06 ^a^*	9.06 ± 0.10 ^a^*	0.13 ± 0.05 ^a^*	66.64 ± 0.21 ^d^*
Massive flowering	18.83 ± 0.03 ^a^*	5.62 ± 0.06 ^b^*	8.93 ± 0.25 ^a^*	0.14 ± 0.02 ^a^*	66.28 ± 0.06 ^cd^*
End of blooming	20.01 ± 0.18 ^b^*	5.14 ± 0.23 ^a^*	8.88 ± 0.11 ^a^*	0.47 ± 0.00 ^a^*	65.55 ± 0.03 ^b^*

The results are expressed as mean ± standard deviation of four replicates. ^a,b,c^ Different lowercase letters within the same column indicate statistical differences between dry untreated raw material. ^a,b,c,d,^* Different lowercase letters with asterisk in the same column indicate statistical differences between residues after CO_2_ extraction. Significant differences were measured using one-way ANOVA (Tukey’s test at the 95% confidence level).

**Table 4:** Total phenolic content and antioxidant capacity of solid plant material before and after SFE-CO_2_.

Samples	TPC, mg GAE/g DW	ABTS, mg TE/g DW	CUPRAC, mg TE/g DW
Intensive growth			
Starting plant material	_A_ 66.54 ± 1.8 ^c^*	_A_ 229.7 ± 3.1 ^b^*	_A_ 245.2 ± 5.3 ^e^*
SFE-CO_2_ residue	_B_ 71.75 ± 1.4 ^c^	_B_ 290.8 ± 4.1 ^c^	_B_ 264.1 ± 7.0 ^d^
Budding phase			
Starting plant material	_A_ 92.10 ± 0.59 ^d^*	_A_ 279.5 ± 3.4 ^c^*	_A_ 185.1 ± 6.8 ^b^*
SFE-CO_2_ residue	_A_ 93.80 ± 0.60 ^d^	_B_ 300.4 ± 1.5 ^d^	_B_ 206.5 ± 2.1 ^c^
Beginning of flowering			
Starting plant material	_A_ 57.91 ± 1.8 ^b^*	_A_ 227.2 ± 2.4 ^b^*	_A_ 195.2 ± 4.7 ^c^*
SFE-CO_2_ residue	_B_ 67.78 ± 2.5 ^b^	_B_ 255.6 ± 5.0 ^b^	_A_ 195.6 ± 7.0 ^b^
Massive flowering			
Starting plant material	_A_ 92.12 ± 2.3 ^d^*	_A_ 282.9 ± 5.3 ^c^*	_A_ 219.5 ± 4.2 ^d^*
SFE-CO_2_ residue	_B_ 100.7 ± 1.8 ^e^	_B_ 372.9 ± 2.6 ^e^	_B_ 269.9 ± 6.3 ^e^
End of blooming			
Starting plant material	_A_ 37.06 ± 0.63 ^a^*	_A_ 123.9 ± 1.7 ^a^*	_A_ 159.1 ± 4.0 ^a^*
SFE-CO_2_ residue	_B_ 54.59 ± 0.45 ^a^	_B_ 231.7 ± 4.1 ^a^	_B_ 180.3 ± 2.2 ^a^

The results are expressed as mean ± standard deviation of four replicates. ^a,b,c,d,e,^* Different lowercase letters with asterisk in the same column indicate statistical differences between dry untreated raw material. ^a,b,c,d,e^ Different lowercase letters within the same column indicate statistical differences between residues after SFE-CO_2_. _A,B_ Different letters within the same column indicate statistical differences between each sample before and after SFE-CO_2_. Significant differences are measured according to Tukey’s test at the 95% confidence level.

**Table 5:** Total phenolic content and antioxidant capacity of *D. canadense* leaves polar extracts obtained by pressurized liquid extraction with acetone, ethanol, and water.

PLE Extracts	TPC, mg GAE/gE	TPC, mg GAE/g DW	ABTS, mg TE/gE	ABTS, mg TE/g DW	CUPRAC, mg TE/gE	CUPRAC, mg TE/g DW	ORAC, mg TE/gE	ORAC, mg TE/g DW
Intensive growth								
PLE-Ac	_B_ 169.7 ± 0.7 ^c^	_A_ 5.90 ± 0.02 ^d^	_B_ 817.6 ± 3.80 ^d^	_A_ 28.45 ± 0.13 ^d^	_B_ 535.9 ± 12.85 ^d^	_A_ 18.65 ± 0.44 ^d^	_B_ 1391 ± 103 ^b^	_A_ 43.56 ± 3.2 ^c^
PLE-EtOH	_C_ 213.0 ± 1.6 ^c^	_B_ 18.63 ± 0.15 ^b^	_C_ 849.9 ± 3.80 ^c^	_B_ 74.28 ± 0.33 ^c^	_C_ 669.4 ± 2.99 ^c^	_C_ 58.50 ± 0.26 ^c^	_C_ 1708 ± 42.3 ^a^	_C_ 149.3 ± 3.7 ^b^
PLE-W	_A_ 132.5 ± 0.8 ^c^	_C_ 21.35 ± 0.13 ^c^	_A_ 590.3 ± 4.31 ^d^	_C_ 94.08 ± 0.39 ^d^	_A_ 353.0 ± 0.66 ^c^	_B_ 51.49 ± 0.10 ^c^	_A_ 688.4 ± 16.7 ^c^	_B_ 21.35 ± 0.13 ^d^
Budding phase								
PLE-Ac	_B_ 178.2 ± 1.1 ^d^	_A_ 5.57 ± 0.03 ^c^	_B_ 810.1 ± 3.8 ^c^	_A_ 25.35 ± 0.11 ^c^	_B_ 600.2 ± 7.1 ^e^	_A_ 18.78 ± 0.22 ^d^	_B_ 1484 ± 30 ^b^	_A_ 46.47 ± 0.96 ^d^
PLE-EtOH	_C_ 259.7 ± 1.7 ^d^	_C_ 33.03 ± 0.22 ^c^	_C_ 975.3 ± 3.8 ^d^	_C_ 124.1 ± 0.48 ^d^	_C_ 853.3 ± 11.9 ^e^	_C_ 108.54 ± 1.5 ^d^	_C_ 1882 ± 75 ^a^	_C_ 239.5 ± 9.6 ^d^
PLE-W	_A_ 169.4 ± 0.07 ^e^	_B_ 28.39 ± 0.11 ^d^	_A_ 672.3 ± 4.3 ^e^	_B_ 111.2 ± 0.71 ^c^	_A_ 347.8 ± 1.1 ^d^	_B_ 58.53 ± 0.19 ^e^	_A_ 855.9 ± 28 ^d^	_B_ 141.58 ± 5.8 ^e^
Beginning of flowering								
PLE-Ac	_B_ 161.4 ± 1.7 ^b^	_A_ 4.71 ± 0.05 ^b^	_B_ 751.2 ± 3.8 ^b^	_A_ 21.93 ± 0.11 ^b^	_B_ 347.6 ± 3.4 ^a^	_A_ 10.15 ± 0.10 ^a^	_B_ 1227 ± 40 ^a^	_A_ 35.84 ± 1.1 ^b^
PLE-EtOH	_C_ 205.2 ± 1.8 ^b^	_C_ 18.72 ± 0.17 ^b^	_C_ 772.8 ± 1.4 ^b^	_C_ 70.48 ± 0.13 ^b^	_C_ 597.9 ± 14 ^b^	_C_ 54.53 ± 1.3 ^b^	_C_ 1693 ± 34 ^a^	_A_ 154.3 ± 3.9 ^a^
PLE-W	_A_ 100.7 ± 0.19 ^b^	_B_ 13.99 ± 0.02 ^b^	_A_ 349.1 ± 1.9 ^b^	_B_ 48.48 ± 0.26 ^b^	_A_ 241.0 ± 2.1 ^b^	_B_ 33.48 ± 0.28 ^b^	_A_ 427.9 ± 27 ^a^	_B_ 37.40 ± 2.42 ^b^
Massive flowering								
PLE-Ac	_B_ 184.3 ± 2.1 ^e^	*_A_* 6.32 ± 0.07 ^e^	_B_ 829.2 ± 4.9 ^e^	_A_ 28.44 ± 0.17 ^d^	_B_ 415.9 ± 3.6 ^c^	_A_ 14.26 ± 0.12 ^c^	_C_ 1952 ± 27 ^c^	_A_ 66.96 ± 0.93 ^e^
PLE-EtOH	_C_ 282.1 ± 2.7 ^d^	*_C_* 38.63 ± 0.08 ^d^	_C_ 1010 ± 1.4 ^e^	_C_ 149.5 ± 0.21 ^e^	_C_ 735.7 ± 3.6 ^d^	_C_ 108.8 ± 0.53 ^d^	_B_ 1756 ± 19 ^a^	_C_ 259.9 ± 2.8 ^bc^
PLE-W	_A_ 141.5 ± 0.19 ^d^	*_B_* 22.86 ± 0.03 ^d^	_A_ 590.2 ± 2.4 ^c^	_B_ 91.81 ± 0.41 ^e^	_A_ 242.7 ± 2.4 ^d^	_B_ 91.81 ± 0.41 ^d^	_A_ 496.2 ± 14 ^b^	_B_ 80.16 ± 2.52 ^c^
End of blooming								
PLE-Ac	_B_ 141.7 ± 1.4 ^a^	_A_ 3.28 ± 0.03 ^a^	_B_ 539.6 ± 2.8 ^a^	_B_ 13.48 ± 0.06 ^a^	_B_ 475.9 ± 8.5 ^c^	_A_ 11.04 ± 0.19 ^b^	_B_ 1139 ± 78 ^a^	_A_ 26.44 ± 1.8 ^a^
PLE-EtOH	_C_ 173.3 ± 1.5 ^a^	_C_ 16.36 ± 0.14 ^a^	_C_ 617.6 ± 2.4 ^a^	_C_ 58.30 ± 0.23 ^a^	_C_ 551.4 ± 6.7 ^a^	_C_ 52.05 ± 0.64 ^a^	_C_ 1672 ± 64 ^a^	_C_ 157.9 ± 6.1 ^c^
PLE-W	_A_ 90.07 ± 0.09 ^a^	_B_ 11.00 ± 0.01 ^a^	_A_ 319.2 ± 3.8 ^a^	_A_ 39.00 ± 0.46 ^a^	_A_ 185.1 ± 2.0 ^a^	_B_ 22.69 ± 0.24 ^a^	_A_ 427.9 ± 27 ^a^	_B_ 37.40 ± 2.4 ^a^

Different superscript letters within the same column indicate statistical differences between different polarity extracts in same extract growth period. Different subscript letters within the column indicate statistical differences between different botanical periods within same solvent (one-way ANOVA, Tukey’s test, 95% confidence level). PLE; pressurized liquid extraction, Ac: acetone, W: water, and EtOH: ethanol, E: extract.

**Table 6:** Phytochemical characterization of semi-polar and polar extracts from *D. canadense* leaves obtained by sequential pressurized liquid extraction with acetone, ethanol, and water.

Peak No.	R_t_	M/Z [M – H]^−^	Suggested Formula	Tentative Identification	PLE-Ac	PLE-EtOH	PLE-W	MS/MS Fragments
1	09	179.0563	C_6_H_11_O_6_	Monosaccharide	+	+	-	-
2	1.0	341.1093	C_12_H_21_O_11_	Dihexose	+	+	-	-
3	1.1	387.1146	C_13_H_23_O_13_	Unknown disaccharide	+	+	+	-
4	1.4	117.0191	C_4_H_5_O_4_	Succinic acid ^a,b,d^	+	+	-	-
5	1.2	133.0141	C_4_H_5_O_5_	Malic acid ^a,b^	+	+	+	-
6	3.1	353.1130	C_16_H_17_O_9_	Caffeoylquinic acid isomer	+	+	+	-
7	3.3	371.1056	C_16_H_19_O_10_	Unidentified	+	+	-	-
8	3.9	337.0930	C_16_H_17_O_8_	Coumaroylquinic acid isomers ^d^	+	+	+	-
9	4.1	289.0718	C_15_H_13_O_6_	Catechin ^a,b,c,d^	+	+	+	151.0396; 245.0809; 165.0188; 123.0450
10	4.6	353.0875	C_16_H_17_O_9_	Chlorogenic acid ^a,b,c,d^	+	+	+	135.0450; 179.0350; 191.0560
11	5.7	431.1915	C_20_H_31_O_10_	Unidentified ^a^	+	+	+	-
12	5.9	337.0930	C_16_H_17_O_8_	Coumaroylquinic acid isomers ^d^	+	+	+	-
13	6.2				+	+	-	-
14	6.4				+	+	-	-
15	6.6	579.1359	C_26_H_27_O_15_	Unidentified flavonoid diglycoside ^c^	+	+	+	357.0601, 429.0823, 459.0931
16	6.8	447.093	C_21_H_19_O_11_	Homoorientin ^b,c,d^	+	+	+	429.0824; 285.0406; 297.0408; 327.0513; 357.0620
17	7.2	563.1412	C_26_H_27_O_14_	Unidentified C-glycosyl flavones ^c,d^	+	+	+	353.0664, 383.0771, 443.0985, 473.1086, 545.1294
18	7.4	447.093	C_21_H_19_O_11_	Orientin ^b,d^	+	+	+	-
19	7.7	563.1415	C_26_H_27_O_14_	Apigenin-6-C-glucosyl-2′′-O-xyloside/Apigenin-8-C-glucosyl-2′′-O-xyloside ^c,d^	+	+	+	293.0455, 413.0879
20	7.9	609.1461	C_27_H_29_O_16_	Rutin ^b,c,d^	+	+	+	300.0272
21	8.1	431.192	C_21_H_19_O_10_	Vitexin ^b,c,d^	+	+	+	311.0559; 341.0664
22	8.5	463.088	C_21_H_19_O_12_	Quercetin 3-O-glucoside ^c^	+	+	+	-
23	8.8	447.0935	C_21_H_19_O_11_	Luteolin-7-O-glucoside ^b,d^	+	+	+	-
24	9.0	593.1511	C_27_H_29_O_15_	Kaempferol-3-O-rutinoside ^a,c,d^	+	+	-	285.0405
25	9.4	593.1513	C_27_H_29_O_15_	C-glycosyl flavones ^d^	+	+	+	-
26	9.7	447.0933	C_21_H_19_O_11_	Quercitrin ^b,d^	+	+	+	-
27	10.2	447.0936	C_21_H_19_O_11_	Astragalin ^c,d^	+	-	-	151.0033; 285.0389
28	11.1	623.1479	C_31_H_27_O_14_	Unidentified ^a^	+	-	-	-
29	11.7	301.0353	C_15_H_9_O_7_	Quercetin ^a^	+	-	-	-
30	12.3	285.0401	C_15_H_9_O_6_	Luteolin ^a^	+	-	-	-
31	1.3	683.2254	C_24_H_43_O_22_	Unknown sugar ^d^	-	+	-	-
32	1.2	191.0141	C_7_H_12_O_6_	Quinic acid ^a,b^	+	+	+	-
33	1.3	191.0196	C_6_H_7_O_7_	Citric acid ^a,b^	-	+	+	-
34	2.2	153.0190	C_7_H_5_O_4_	Gentisic acid ^a,d^	-	-	+	-
35	2.4	315.0794	C_13_H_15_O_9_	Gentisoyl glucoside ^a^	-	+	+	-
36	2.6	355.1030	C_16_H_19_O_9_	Gentiopicrin ^a^	-	+	+	-
37	6.9	163.0473	C_9_H_7_O_3_	*p*-coumaric acid ^b^	+	+	+	-

+: Detected; -: not detected, ^a^ Confirmed by parent ion mass using the free chemical databases (ChemSpider (CS), PubChem (PC); ^b^ Confirmed by a standard; ^c^ Confirmed by MS/MS; ^d^ Confirmed by a reference.

**Table 7:** Quantification of organic acids and flavonoids in PLE extracts of *D. canadense* across vegetation periods (mg/100 g DW).

VP	Organic Acids					Flavones			Flavonols			Flavanol
	Citric Acid	Quinic Acid	Malic Acid	*p*-Coum. Acid	Chl. Acid	Orientin	Lut.-7-O-Glucoside	Vitexin	Quercitrin	Rutin	Quer. 3-Glucoside	Catechin
	Acetone extracts											
IG	ND	1.33 ± 0.05 ^ab^	0.57 ± 0.41 ^a^	0.97 ± 0.11 ^a^	0.48 ± 0.02 ^c^	15.11 ± 1.49 ^c^	5.32 ± 0.08 ^c^	448.2 ± 0.62 ^d^	2.13 ± 0.14 ^a^	24.14 ± 0.66 ^b^	1.67 ± 0.02 ^a^	15.79 ± 0.38 ^b^
BP	ND	2.08 ± 0.17 ^c^	0.49 ± 0.03 ^a^	0.95 ± 0.01 ^a^	0.34 ± 0.01 ^b^	22.96 ± 0.14 ^d^	6.26 ± 0.10 ^d^	325.3 ± 8.9 ^c^	18.31 ± 0.13 ^b^	34.85 ± 0.14 ^c^	5.71 ± 0.13 ^b^	20.66 ± 0.25 ^c^
BF	ND	1.63 ± 0.11 ^b^	1.05 ± 0.07 ^b^	1.48 ± 0.06 ^c^	0.24 ± 0.02 ^b^	11.18 ± 0.23 ^b^	2.69 ± 0.43 ^a^	142.4 ± 5.0 ^b^	131.8 ± 1.91 ^d^	56.94 ± 0.04 ^d^	30.04 ± 0.82 ^d^	16.32 ± 0.11 ^b^
MF	ND	1.50 ± 0.09 ^b^	0.81 ± 0.12 ^ab^	1.27 ± 0.06 ^b^	0.22 ± 0.06 ^b^	13.90 ± 0.05 ^bc^	3.93 ± 0.21 ^b^	141.8 ± 8.4 ^b^	63.62 ± 3.37 ^c^	36.59 ± 1.4 ^c^	17.08 ± 0.03 ^c^	23.75 ± 0.21 ^d^
EB	ND	1.01 ± 0.03 ^a^	0.52 ± 0.04 ^a^	0.91 ± 0.06 ^a^	0.15 ± 0.00 ^a^	8.29 ± 0.45 ^a^	2.01 ± 0.03 ^a^	120.4 ± 2.3 ^a^	17.75 ± 0.13 ^b^	15.91 ± 0.04 ^a^	4.61 ± 0.13 ^b^	10.13 ± 0.15 ^a^
	Ethanol extracts											
IG	3.64 ± 0.81 ^ab^	21.93 ± 1.6 ^a^	3.84 ± 0.15 ^a^	1.51 ± 0.11 ^a^	16.56 ± 0.11 ^c^	14.52 ± 0.57 ^a^	5.98 ± 0.21 ^b^	160.2 ± 4.2 ^c^	1.79 ± 0.28 ^a^	48.52 ± 4.1 ^a^	2.52 ± 0.24 ^a^	44.21 ± 1.6 ^c^
BP	2.65 ± 0.25 ^ab^	19.45 ± 1.3 ^a^	4.92 ± 0.04 ^a^	1.55 ± 0.13 ^a^	1.42 ± 0.07 ^a^	17.56 ± 0.28 ^b^	6.60 ± 0.43 ^b^	100.2 ± 1.6 ^b^	13.06 ± 0.40 ^b^	55.88 ± 2.4 ^ab^	7.84 ± 0.05 ^b^	56.42 ± 0.01 ^d^
BF	4.01 ± 0.90 ^b^	19.20 ± 1.9 ^a^	13.31 ± 1.5 ^b^	1.62 ± 0.01 ^a^	1.41 ± 0.06 ^a^	13.96 ± 1.2 ^a^	3.53 ± 0.16 ^a^	88.74 ± 2.6 ^a^	91.67 ± 2.5 ^e^	108.4 ± 1.9 ^c^	36.41 ± 1.1 ^e^	32.64 ± 1.9 ^a^
MF	2.34 ± 0.09 ^a^	41.38 ± 0.49 ^c^	18.38 ± 1.3 ^c^	2.00 ± 0.19 ^b^	2.35 ± 0.22 ^b^	20.01 ± 1.1 ^c^	6.62 ± 0.05 ^b^	117.2 ± 1.2 ^b^	48.12 ± 0.68 ^d^	104.2 ± 0.5 ^c^	25.94 ± 0.86 ^d^	56.01 ± 0.18 ^d^
EB	2.51 ± 0.18 ^a^	35.54 ± 3.1 ^b^	26.83 ± 1.2 ^d^	1.76 ± 0.03 ^b^	1.28 ± 0.26 ^a^	14.21 ± 0.20 ^a^	3.65 ± 0.11 ^a^	99.67 ± 8.8 ^ab^	18.36 ± 1.1 ^c^	62.20 ± 3.6 ^b^	12.66 ± 0.38 ^c^	37.38 ± 1.8 ^b^
	Water extracts											
IG	152.2 ± 1.4 ^a^	76.19 ± 0.63 ^d^	142.6 ± 1.5 ^a^	10.35 ± 0.24 ^d^	11.35 ± 0.11 ^d^	4.06 ± 0.27 ^b^	2.91 ± 0.21 ^cd^	13.80 ± 1.25 ^b^	0.65 ± 0.04 ^a^	6.59 ± 0.67 ^a^	1.15 ± 0.09 ^a^	46.04 ± 0.4 ^e^
BP	193.3 ± 4.5 ^a^	78.09 ± 0.52 ^d^	173.9 ± 4.1 ^b^	7.85 ± 0.54 ^b^	11.07 ± 0.11 ^d^	6.26 ± 0.94 ^c^	3.33 ± 0.22 ^d^	16.41 ± 0.11 ^c^	1.59 ± 0.40 ^a^	18.44 ± 0.30 ^b^	2.32 ± 0.10 ^c^	41.17 ± 0.22 ^d^
BF	433.9 ± 32 ^b^	51.94 ± 0.53 ^b^	358.3 ± 6.8 ^e^	8.15 ± 0.06 ^b^	8.97 ± 0.03 ^c^	4.49 ± 0.46 ^b^	2.65 ± 0.22 ^c^	12.33 ± 0.72 ^b^	9.75 ± 0.62 ^c^	30.74 ± 2.09 ^c^	7.47 ± 0.06 ^e^	24.07 ± 0.06 ^b^
MF	627.6 ± 9.8 ^c^	64.04 ± 2.1 ^c^	338.6 ± 1.0 ^d^	9.63 ± 0.39 ^c^	7.69 ± 0.56 ^b^	2.94 ± 0.19 ^a^	1.79 ± 0.07 ^b^	4.56 ± 1.10 ^a^	4.32 ± 0.29 ^b^	4.91 ± 0.27 ^a^	3.45 ± 0.19 ^d^	32.16 ± 1.0 ^c^
EB	438.7 ± 5.3 ^b^	41.60 ± 0.78 ^a^	253.1 ± 3.7 ^c^	5.42 ± 0.28 ^a^	4.12 ± 0.17 ^a^	2.41 ± 0.84 ^a^	1.10 ± 0.03 ^a^	5.19 ± 0.23 ^a^	1.41 ± 0.28 ^a^	5.18 ± 1.47 ^a^	2.01 ± 0.11 ^b^	22.04 ± 1.0 ^a^

The growth periods shown in the table are abbreviated as follows: IG—intensive growth, BP—budding phase, BF—beginning of flowering, MF—mass flowering, and EB—end of blooming, VP—vegetation periods, *p*-Coum—*p*-coumaric, Lut—luteolin, Quer—quercetin, Chl—chlorogenic acid. ND—not determined. Different lowercase letters indicate significant differences between growth periods for the same compound, according to Tukey’s test at the 95% confidence level.

## Data Availability

Data available from the authors upon request.

## Supplementary Materials

The following supporting information can be downloaded at: https://www.mdpi.com/article/10.3390/molecules31030528/s1. Methods Section S1: In Vitro Antioxidant Capacity of Extracts and Solid Residues. Table S1: Sum of each compound extracted with acetone, ethanol, and water (expressed as mg TE/100 g DW) at each growth stage
