# Supplementary material for: Consecutive Recovery of Bioactive Substances from Desmodium canadense at Different Plant Vegetation Phases by Green Extraction with Supercritical CO2 and Increasing Polarity Pressurized Liquids
Source: Molecules. 2026 Feb 3;31(3):528. doi: 10.3390/molecules31030528 (PMC12899499; doi:10.3390/molecules31030528)
Supplement: Supplementary file 1 [file molecules-31-00528-s001.zip › molecules-4111413-supplementary.pdf]

Supplementary Materials

Consecutive recovery of bioactive substances from *Desmodium canadense* at different plant vegetation phases by green extraction with supercritical CO_2 and increasing polarity pressurized liquids

Sana Abbas[1], Milda Pukalskienė[1], Laura Jūrienė, Ona Ragažinskienė[2], Petras Rimantas Venskutonis[1]*

[1] Kaunas University of Technology, Department of Food Science and Technology, Radvilėnų pl. 19, Kaunas LT-50254, Lithuania

[2] Kaunas Botanical Garden of Vytautas Magnus University, Ž.E. Žilibero str. 6, Kaunas LT-46324, Lithuania

*Correspondence: Petras Rimantas Venskutonis rimas.venskutonis@ktu.lt

S1. In Vitro Antioxidant Capacity of Extracts and Solid Residues

S1.1. Oxygen Radical Absorbance Capacity (ORAC) Assay

The ORAC of PLE extracts was determined using fluorescein as the fluorescent probe according to Prior et al. (2003). Fluorescein stock solution was prepared in 75 mM phosphate-buffered saline (PBS; pH 7.4). In a 96-well opaque microplate, 25 µL of sample or methanol (blank) was mixed with 150 µL fluorescein solution. The plate was covered, incubated at 37 °C for 15 min, then 240 mmol/L AAPH (prepared freshly in PBS) was added as a peroxyl radical generator. The plate was placed again into the fluorescent reader and measurements were started. AAPH and fluorescein solutions are always prepared freshly before measurements. The plate was automatically shaken before each measure and the fluorescence was recorded every 1min × 1.1 cycle and a total of 120 cycles, with excitation (485-P) and emission (520-P) filters. Raw data were imported to Excel 2003 (Microsoft, Roselle, IL) from the Mars software (BMG Labtech GmbH, Offenburg, Germany), curves (fluorescence versus time) were normalized and the area under the decay curve (AUC) was recorded as:

$AUC = \sum_{i=1}^{i=150} \frac{f\,i}{f\,0}$ Where, fi is the fluorescence recorded at i time and f0 is fluorescence at 0 minutes. Various concentrations of Trolox (6-hydroxy-2,5,7,8-tetramethylchroman-2-carboxylic acid) between 0–500 mol/L in PBS were prepared to develop calibration curve and the trolox equivalent antioxidant capacity ($TEAC_{ORAC}$) of extracts was calculated using dose-response curves for Trolox ($y = 0.1394x - 0.7395$, R2 = 0.9926)

S1.2. $ABTS^{\bullet+}$ Scavenging Assay

ABTS radical scavenging capacity was measured following Re et al. (1999) with modifications. A 75 mmol/L PBS (pH 7.4) was prepared by dissolving 8.18 g NaCl, 0.15 g KCl, 0.27 g KH_2PO_4, and 1.42 g Na_2HPO_4 in 1 L distilled water. $ABTS^{\bullet+}$ stock solution was made by mixing 50 mL ABTS (2 mmol/L

in PBS) with 200 μL $K_2S_2O_8$ (70 mmol/L) and kept in the dark for 15–16 h at room temperature. The working solution was diluted with PBS to an absorbance of 0.700 ± 0.010 at 734 nm. For the solution assay, 1500 μL of $ABTS^{•+}$ working solution was mixed with 25 μL sample or methanol (blank), vortexed, kept in the dark for 2 h, and absorbance was measured at 734 nm using an Orion AquaMate spectrophotometer (Thermo Scientific, USA). Trolox (0–1500 μmol/L) was used for calibration (y = 0.0604x + 1.0413, R◎= 0.9997).

For the QUENCHER format, 10 mg sample or blank (cellulose) was mixed with 1500 μL $ABTS^{•+}$ solution and 25 μL methanol, vortexed for 15 s, shaken (250 rpm, 2 h), centrifuged (4500 rpm, 5 min), and absorbance of the supernatant was read at 730 nm. Calibration was performed with Trolox (y = 0.0667x + 1.1957, R◎= 0.9991).

S1.3. Cupric Reducing Antioxidant Capacity (CUPRAC) Assay

CUPRAC was measured using the method of Apak et al. (2007) with modifications. Solutions were prepared as follows: 1 mM $CuCl_2$ (0.4262 ± 0.002 g $CuCl_2 \cdot H_2O$ in 250 mL water), 7.5 mM neocuproine (0.0039 ± 0.002 g in 25 mL ethanol, 96%), and NH_4Ac buffer (19.27 ± 0.002 g in 250 mL water, pH 7). For the solution assay, 0.4 mL each of $CuCl_2$, neocuproine, and NH_4Ac buffer was added to 0.4 mL sample or blank, incubated in the dark for 30 min, and absorbance was measured at 450 nm. Trolox was used for calibration (y = 0.0035x + 0.007, R◎= 0.9977).

For the QUENCHER assay, 10 mg sample was mixed with 0.4 mL water and the same reagent volumes, vortexed 15 s, shaken (2 h), centrifuged, and absorbance measured at 450 nm. Calibration was with Trolox (25–200 μmol/L).

S2. Total Phenolic Content (TPC) by Folin–Ciocalteu Assay

TPC was determined using the method of Singleton et al. (1999) with modifications. The Folin Ciocalteu reagent was diluted 1:9 with distilled water, and Na_2CO_3 solution was prepared (75 g/L). Extracts were dissolved in their respective solvents. For the solution assay, 0.15 mL extract was mixed with 0.75 mL Folin Ciocalteu reagent and 0.6 mL Na_2CO_3, kept in the dark for 2 h, and absorbance read at 760 nm. Gallic acid calibration (0–80 μg/mL) was used (y = 0.0118x + 0.0092, R◎= 0.9977).

For QUENCHER, 10 mg sample (solid material before and after SFE-CO_2) or blank (cellulose) was mixed with Folin–Ciocalteu and Na_2CO_3, shaken in the dark (250 rpm, 2 h), centrifuged (4500 × g, 5 min), and absorbance measured at 760 nm (Orion AquaMate spectrophotometer). Calibration was with gallic acid (y = 0.0113x + 0.0131, R◎= 0.9994).

Table S1. Sum of each compound extracted with acetone, ethanol, and water (expressed as mg TE/100 g DW) at each growth stage.

**Sample**	**Intensive growth**	**Budding phase**	**Beginning of flowering**	**Massive flowering**	**End of blooming**
Citric acid	156.5±2.27	196.8±1.33	438.8±32	631.3±5.9	437.7±5.1
Quinic acid	99.44±2.05	99.62±2.23	72.58±2.32	106.9±1.48	78.16±4.02
Malic acid	146.9±1.4	179.5±4.3	372.8±5.3	357.7±1.1	280.4±5.1
p-coumaric acid	12.83±0.45	10.46±0.67	11.25±0.01	12.90±0.26	8.095±0.26
Chlorogenic acid	28.38±0.20	12.82±0.01	17.84±0.08	11.51±0.78	5.546±0.09
Orientin	33.70±1.80	46.78±0.52	29.63±1.51	36.85±0.82	24.90±0.80
Luteolin -7-o-oglucoside	14.21±0.09	16.19±0.32	8.872±0.36	12.35±0.08	6.765±0.17
Vitexin	622.2±5.6	441.9±7.1	243.5±1.0	263.5±3.3	225.2±1.5
Quercitrin	4.568±0.009	32.970.83	233.2±0.01	116.1±2.4	37.53±0.64
Rutin	79.25±2.68	109.2±2.25	196.1±0.54	145.6±1.63	83.293.12
Quercetin 3 glucoside	5.3390.36	15.87±0.18	73.92±0.30	46.47±0.64	19.28±36
Catechin	106.0±0.9	118.2±0.03	73.042.63	111.9±1.06	69.56±0.63
